# Amino Acid Uptake in Arbuscular Mycorrhizal Plants

**DOI:** 10.1371/journal.pone.0047643

**Published:** 2012-10-17

**Authors:** Matthew D. Whiteside, Maria O. Garcia, Kathleen K. Treseder

**Affiliations:** 1 Biology Department and Institute for Species at Risk and Habitat Studies, University of British Columbia Okanagan, Kelowna, British Columbia, Canada; 2 Forest Ecosystems and Society, Oregon State University, Corvallis, Oregon, United States of America; 3 Department of Ecology and Evolutionary Biology, University of California Irvine, Irvine, California, United States of America; Max Planck Institute for Chemical Ecology, Germany

## Abstract

We examined the extent to which arbuscular mycorrhizal (AM) fungi root improved the acquisition of simple organic nitrogen (ON) compounds by their host plants. In a greenhouse-based study, we used quantum dots (fluorescent nanoparticles) to assess uptake of each of the 20 proteinaceous amino acids by AM-colonized versus uncolonized plants. We found that AM colonization increased uptake of phenylalanine, lysine, asparagine, arginine, histidine, methionine, tryptophan, and cysteine; and reduced uptake of aspartic acid. Arbuscular mycorrhizal colonization had the greatest effect on uptake of amino acids that are relatively rare in proteins. In addition, AM fungi facilitated uptake of neutral and positively-charged amino acids more than negatively-charged amino acids. Overall, the AM fungi used in this study appeared to improve access by plants to a number of amino acids, but not necessarily those that are common or negatively-charged.

## Introduction

Arbuscular mycorrhizal fungi are well known for their ability to supply plants with scarce inorganic nutrients, such as ammonium, nitrate, and phosphate, while obtaining carbon (C) from the plant host [1]. In a recent greenhouse study, 75% of the nitrogen (N) found in *Zea maize* shoots was transferred to the plants via the AM fungus *Glomus aggregatum* [2]. Despite being restricted to obligate biotrophy and requiring plant C to grow, AM fungi are capable of accessing ON from a few soil sources, including labile amino acids [3], N-containing polysaccharides [4], [5], and decaying plant material [6], [7]. However, because uncolonized plant roots can also access ON, such as labile amino acids and relatively large proteins from soils [8], [9], the extent to which AM fungi improve plant uptake of soil ON is not well-resolved.

Currently, there is little agreement about which types of ON are preferred by AM fungi. In a recent review, Talbot and Treseder [10] hypothesized that relatively abundant (i.e., most often incorporated into proteins), easy to break down (i.e., not aromatic), and N-rich amino acids are likely preferred by mycorrhizal fungi. This hypothesis was based on the extent to which 26 mycorrhizal species (22 ectomycorrhizal, 2 ericoid, and 2 AM from 20 independent studies) were able to access amino acids as their sole source of N. Previously, Jones and Darrah [11] had proposed that neutral amino acids should be taken up by plants more readily than negatively or positively charged amino acids, owing to faster diffusion rates by the neutral amino acids. Mycorrhizal fungi might be influenced by similar mechanisms.

We tested the suggestions of Talbot and Treseder [10] and Jones and Darrah [11] in a laboratory experiment. Specifically, we hypothesize that AM fungi will improve plant uptake of (1) abundant amino acids more than rarer amino acids, (2) aliphatic amino acids more than aromatic amino acids, (3) amino acids with relatively high N content more than those with relatively low N content, and (4) neutrally-charged more than positively- or negatively-charged amino acids. To test these hypotheses, we used quantum dots (QDs), which are fluorescent nanoscale semiconductors that can be used to trace ON uptake into AM fungi and plants [4], [12]. We covalently labeled the amino groups of 20 proteinaceous amino acids with carboxyl terminating QDs to quantify amino acid uptake by plants colonized by AM fungi versus those that remained uncolonized.

## Methods

### QD Conjugation

QD-labeled amino acids were prepared according to a modified Whiteside et al. [4] method. Specifically, QDs were conjugated to the amino groups of each amino acid. Commercial green (530 nm emission) carboxyl terminated QDs (3 nm diameter) were purchased from ViveNano (Toronto, Canada). Stock QD solutions were conjugated with individual amino acids (either Ala, Arg, Asn, Asp, Cys, Gln, Glu, Gly, His, Ile, Leu, Lys, Met, Phe, Pro, Ser, Thr, Try, Tyr, or Val) in a 33∶1 ratio (substrate: QD) using the binding activator1-ethyl-3-(3-dimethylaminopropyl) carbodiimide hydrochloride (EDC). After 2 h of conjugation, each solution underwent dialysis against 2 L of sterile water.

### Plant Uptake

To determine ON uptake by plants, QD-labeled amino acids were incubated with aseptic cultures of AM and uninoculated Sudan grass seedlings (*Sorghum bicolor*). Individual seedlings were cultivated in 10 ml test tubes covered with 8-ply sterile cheesecloth. Each tube contained 3.5 g autoclaved 1∶1 sand: vermiculite and 1.6 ml half-strength Melin-Norkrans (MMN) liquid media (pH = 7.4). Half of the seedlings were inoculated with 60–80 spores of a mixture of four AM species: *Glomus intraradices*, *Glomus etunicatum*, *Glomus mosseae,* and *Glomus aggregatum.* (Mycorrhizal Applications, Grants Pass, Oregon, USA). To maintain consistent media levels, 1 ml of sterile water was added to each tube every 10 d. After 45 d of growth, 1.5 ml (0.8 µM) of each QD-amino acid treatment was injected into the sand: vermiculite of four AM and four uninoculated seedlings. An additional set of seedlings received QD-controls, which consisted of unbound QDs subjected to the same conditions as labeled conjugates, but lacking amino acid substrates. A separate set of seedlings did not receive any QD injections and were used as no-injection controls. Each treatment was replicated four times. All procedures were conducted using sterile techniques, and samples were routinely checked for contamination. In addition, sand has a low cation exchange capacity.

After 24 h of incubation plant shoots were harvested and dried at 60°C for 48 h. Dry shoots were weighed on an analytical balance. Shoots were homogenized with Zirconia beads in 50 mM (pH 8.0) bicarbonate buffer (100 µl buffer mg^−1^ shoot). Microplate wells were each filled with one 200 µl aliquot homogenized sample.

### Microplate Quantification

QD quantification was performed using a standard 96-well epi-fluorescence microplate reader. Fluorescent intensities (A.U. mg^−1^ plant shoot d^−1^) were determined at 450 nm excitation and 530±10 nm emission. To remove background fluorescence, blank wells and no-injection plant controls were subtracted from each sample. Fluorescent intensities were (A.U. mg^−1^ plant shoot d^−1^) converted to specific uptake rates (nmol QD mg^−1^ plant shoot d^−1^) using a calibration gradient of QD-controls. Total plant uptake (nmol QD plant shoot^−1^ d^−1^) was calculated as the product of specific uptake (nmol QD mg^−1^ plant shoot d^−1^) and dry weight of the total shoot.

### Statistics

The data were not normally distributed, so we performed non-parametric tests. To determine whether AM fungi increased uptake of a given amino acid (QD uptake shoot^−1^ d^−1^), we conducted a series of Kruskal-Wallis tests with uptake of each amino acid as the dependent variable, and mycorrhizal status (AM plants vs. uninoculated plants) as the independent variable. Next, we calculated a Cohen’s *d* effect size for each amino acid:
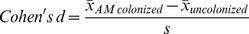



Where 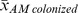 is the mean uptake of a given amino acid in AM colonized plants, 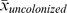 is the mean for uncolonized plants, and *s* is the pooled standard deviation. To test Hypothesis 1, we performed a Spearman ranked correlation between the proportional abundance of each amino acid in proteins (Table 1, from http://www.ncbi.nlm.nih.gov) and effect size of uptake. For Hypothesis 2, we used a Kruskal-Wallis test to compare effect sizes between aromatic and aliphatic amino acids. Effect size of uptake was the dependent variable; aromatic amino acids were represented by phenylalanine, tryptophan, and tyrosine; and aliphatic amino acids were represented by all others. To test Hypothesis 3, we conducted a Spearman ranked correlation between effect size of uptake and the percentage N content by weight of each amino acid (Table 1). For Hypothesis 4, we used a Kruskal-Wallis test to compare effect size of uptake between neutral, negative, and positive amino acids (Table 1), followed by a Kolmogorov-Smirnov Two-Sample test. In all cases, differences were considered significant when P<0.05 and marginally significant when P<0.10.

**Table 1:** Characteristics and effect sizes of AM colonization for amino acids.

Amino acid	%N(by weight)	Abundance(%)†	Charge	Structure	AM effect size(Cohen’s d)
Ala	16%	9%	Neutral	Aliphatic	−0.56
Arg	32%	6%	Positive	Aliphatic	1.65
Asn	21%	4%	Neutral	Aliphatic	1.41
Asp acid	11%	5%	Negative	Aliphatic	−4.23
Cys	12%	1%	Neutral	Aliphatic	10.57
Gln	19%	4%	Neutral	Aliphatic	0.88
Glu acid	10%	6%	Negative	Aliphatic	−0.80
Gly	19%	7%	Neutral	Aliphatic	1.17
His	27%	2%	Neutral	Aliphatic	2.71
Ile	11%	6%	Neutral	Aliphatic	0.68
Leu	11%	10%	Neutral	Aliphatic	−0.38
Lys	19%	5%	Positive	Aliphatic	1.27
Met	9%	2%	Neutral	Aliphatic	2.84
Phe	8%	4%	Neutral	Aromatic	1.21
Pro	12%	5%	Neutral	Aliphatic	0.67
Ser	13%	7%	Neutral	Aliphatic	−0.14
Thr	12%	5%	Neutral	Aliphatic	0.69
Trp	8%	1%	Neutral	Aromatic	3.91
Tyr	14%	3%	Neutral	Aromatic	−0.64
Val	12%	7%	Neutral	Aliphatic	−0.58

† Relative abundance is the percentage of amino acids incorporated into proteins, based on all protein sequences accessible in GenBank (www.ncbi.nlm.nih.gov).

## Results

**Figure 1:**
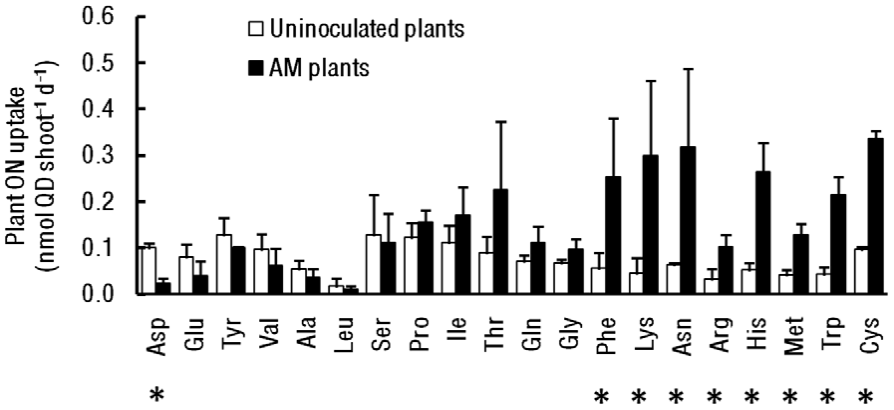
Amino acid uptake by AM and uninoculated plants. Abbreviations for amino acids are standard. Bars are means +1 SE. Asterisks indicate significant pairwise differences between uninoculated and AM-colonized plants (P<0.05).

**Figure 2:**
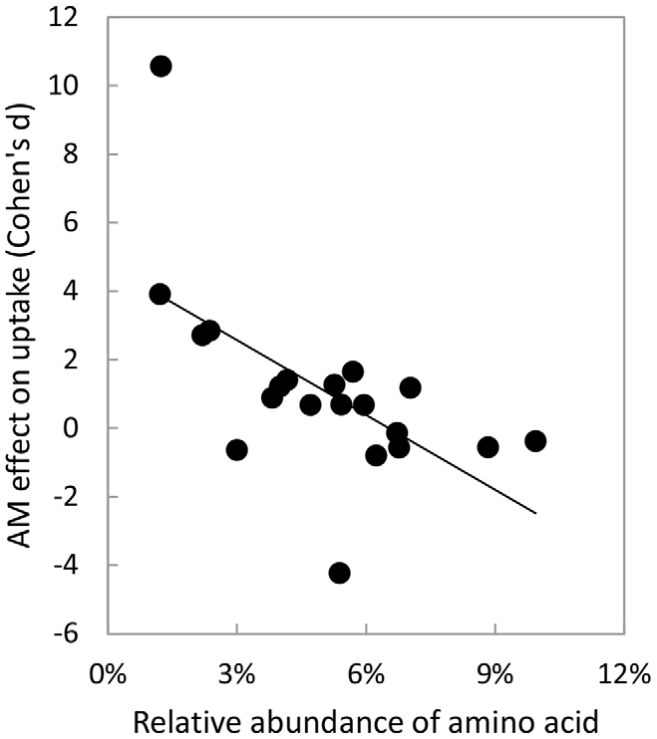
Relationship between the relative abundance of amino acids in proteins and the effect size of AM colonization on plant uptake of amino acids. Relative abundance was determined across all protein sequences accessible in GenBank (www.ncbi.nlm.nih.gov). Each symbol represents one amino acid; line is best-fit. The two variables were significantly negatively related to one another (P = 0.003).

Compared to uncolonized plants, AM plants took up significantly greater amounts of phenylalanine, lysine, asparagine, arginine, histidine, methionine, tryptophan, and cysteine (Fig. 1; P<0.05). In contrast, aspartic acid was taken up less by AM-colonized plants than uncolonized plants (P = 0.03). Colonization status did not significantly influence uptake of any other amino acids. Arbuscular mycorrhizal colonization most strongly influenced uptake of rarer amino acids, which was the opposite pattern predicted for Hypothesis 1 (Fig. 2, r = −0.62, P = 0.003). Effect sizes did not differ significantly between aromatic (1.50±1.32, mean ±SE) and aliphatic amino acids (1.05±0.71), leading us to reject Hypothesis 2 (H = 22.00, P = 0.711). Likewise, Hypothesis 3 was rejected, because N content of amino acids was not significantly correlated with effect size (data not shown, r = 0.12, P = 0.61). Finally, Hypothesis 4 was only partially supported. Neutral, negatively-, and positively-charged amino acids differed marginally significantly from one another (H = 5.93, P = 0.052), and effect sizes for neutral amino acids (1.53±0.69) and positively-charged amino acids (1.46±0.19) were significantly greater than for negatively-charged amino acids (−2.51±1.72, P<0.001 for both). However, neutral and positively-charged amino acids did not differ significantly from one another (P = 0.63).

## Discussion

This study documented that AM fungi improved plant uptake of multiple QD-amino acids: phenylalanine, lysine, asparagine, arginine, histidine, methionine, tryptophan, and cysteine. For these amino acids, the average increase in uptake was four-fold. Nevertheless, it is unclear why AM fungi improved uptake of these amino acids but not others. We rejected each of our hypotheses regarding preferences of AM fungi. Indeed, we observed the opposite pattern predicted by Hypothesis 1; rarer amino acids were taken up more readily by AM fungi. Talbot and Treseder [10] had predicted that more common amino acids should be targeted, in order to increase uptake rates per unit investment in membrane transport proteins. In fact, they found this to be the case when considering studies that were dominated by ectomycorrhizal fungi. If non-AM fungi, such as ectomycorrhizal fungi, target the more common amino acids, then they may compete less with AM fungi for rarer amino acids.

We also observed less AM uptake of negatively-charged amino acids than neutral or positively-charged amino acids. Nitrogen uptake can be influenced by biochemical interactions between the N substrate and the cell surface chemistry [13]–[15]. Rufyikiri [16] found that the affinities of AM inoculated roots for positively-charged substrates were four times larger than those of uninoculated plants. Likewise, AM fungi can improve cation exchange capacities in roots [17]. The faster uptake of positively-charged amino acids may have resulted from these characteristics of AM fungi [18]. In addition, relatively high uptake rates of neutral amino acids could have been due to greater diffusion rates of these amino acids in soil solution.

While it is clear from previous work that AM fungi improve nutrient uptake by plants, few studies have specifically examined ON uptake. In greenhouse studies, AM plants frequently take up more inorganic N and P from soil than uncolonized plants (e.g.,[2], [3], [19], [20]–[25]). Although relatively few corresponding studies have tested ON uptake, similar results have been documented using plant litter [26], [27] and unbound amino acids, including arginine [28], cysteine [2], glycine [3], glutamine [3], and methionine [2]. We did not observe significant uptake of glycine and glutamine in our study, but uptake preferences might vary among AM isolates and host plants.

*Sorghum bicolor* was selected as a model plant for this study based on fast growth rates and ability to host multiple masses of AM fungi. However, this C_4_ grass is an especially efficient biomass accumulator with biochemical and morphological specializations that increase net carbon assimilation at high temperatures [29]. As an agricultural plant inoculated with non-native AM fungi from one genus, it may not adequately represent plants from natural communities with diverse, native AM partners. Future studies might examine the degree to which AM fungi adapt to particular hosts and their nitrogen environments. In addition, the use of QDs may have influenced substrate uptake by increasing mass or volume of the substrate.

We did not determine the extent to which the amino acids might have been transformed before, during, or after uptake. Activities of extracellular enzymes released from plant roots or AM fungi could have altered the structure of amino acids prior to uptake [10]. Likewise, the amino acids could have been transformed within the AM fungi before they were transferred to the host plant. As such, the uptake rates provided here are not specifically for intact amino acids, but rather for the amino group that was directly conjugated to the QD. Thus, the QD uptake rates indicate the rate at which amino acid-derived N is obtained by the host plant. We note that since the microcosms were aseptic, the amino acids could not have been mineralized or otherwise transformed by non-AM microbes.

Given that about 40% of soil N is in the form of amino acids and other proteinaceous material [30], selection pressures to acquire this form of N would presumably be greatest where inorganic N is limiting and particularly efficient plant-fungal combinations have been well established. For example, in northern boreal forests where inorganic N availabilities are relatively low, both plants and their AM fungi, or only the fungus partner, may have evolved greater ON uptake efficiencies relative to AM fungal-plant pairs that adapted to habitats with high inorganic N. Indeed, Whiteside et al. [12] observed greater amino acid uptake by AM fungi in unfertilized versus N-fertilized plots in an Alaskan boreal forest. Evaluating differently adapted plant and fungus partners, alone and in combination, under controlled conditions may reveal a more complete picture of AM fungal contribution to plant ON uptake. QD technology is particularly suitable for these studies, because amino acid N is transferred to plants when bound to a 3 nm diameter QD, providing the potential to trace ON compounds that could be as large as small proteins.
